# Study on the structural characteristics of China's high-speed railway network and its coordination with economic growth based on Fractal theory

**DOI:** 10.1016/j.heliyon.2023.e21398

**Published:** 2023-11-03

**Authors:** Xiaoshan Cai, Shaopei Chen, Xinying Lian

**Affiliations:** a School of Culture Tourism and Geography, Guangdong University of Finance and Economics, Guangzhou, China; b School of Public Administration, Guangdong University of Finance and Economics, Guangzhou, China

**Keywords:** High-speed railway network, Fractal dimension, Spatial heterogeneity, Nonstationarity, China

## Abstract

As one of the modern transportation modes, the high-speed railway network system has been a robust part of the comprehensive transportation system in China. An important topic emerges the exploration and optimization of its structural organization and coordinated relationship with the regional development, including urban form, land use, and economy. Therefore, supported by the integration of geographical information system (GIS) and fractal theory, this paper aims to carry out an investigation and discussion on the structural characteristics, including intensity (density), complexity, nonstationarity, and heterogeneity of the high-speed railway network in China (HSRNC) from the perspective of the whole country and specific regions, i.e., urban agglomerations. Moreover, based on the time-series data of network mileage expansion and economic output analysis, this study aims to evaluate and characterize the coordinated relationships between network development and economic growth in the context of the nationwide area and urban agglomerations. This study aims to explore and promote the spatial structural organization and morphology of the high-speed railway network in China, thus improving the coordinated development with the regional economic growth, for giving a new perspective to the future planning and evolution of the high-speed railway network in China.

## 1 Introduction

The transportation network is a significant spatial network carrier linking the flow of production factors and economic and social activities between different regions. The networking and structuring process of the transportation network is closely related to regional development, including urban expansion, economic growth, environmental change, and land use [[1], [2], [3], [4], [5], [6], [7], [8], [9], [10]]. With the rapid development of modern transportation technologies, the high-speed transportation network system led by high-speed railways, expressways, and airlines, has been promoting and guiding regional development. Considering the high-speed transportation development in the world, the construction of a high-speed railway network has transformed from a mere solution to regional traffic demand to an essential means of guiding and promoting the spatial structural organization of cities and towns in the region, the efficiency of land use and industrial clusters, as well as the economic spatial connections and the functional complementarities between different areas [[11], [12], [13], [14], [15], [16]]. Especially in the emerging country of China, the high-speed railway network system has formed the major arteries to keep the regions in tight spatial (economic) correlation and structuring and networking in the context of its rapid urbanization [[17], [18], [19]]. Consequently, the high-speed railway network in China (HSRNC) development facilitates the formation and reconstruction of the regional structural organization and determines the regional development trend in China [[20], [22], [21]]. Therefore, the exploration and interpretation of the structural characteristics of HSRNC should be deeply explored and discussed, especially for the interaction and adaptive pattern with the coordination of the regional spatial form evolution and economic development. The aim is to construct the strategies and mechanisms to achieve orderly integration, efficient operation, and mutual support between network evolution and regional development.

In the context of regional coordinated development, the study of the correlation between regional transportation network development and economic growth has been a topic of interest for more and more scholars, which focused on the investigation and discussion of the transportation spatial accessibility and economic development adaptability through the applications of some sophisticated theories and models, such as location theory, transportation organization and supply-demand relationship, econometric models and network science (such as a graph, complexity network, spatial syntax, traffic flow, and fractal geometry model) [[23], [24], [25], [26], [27], [28], [29]]. In the current research, the level of accessibility is referred to as a significant index to interpret the structural characteristics of transportation networks and characterize the spatial structural organization of a region by using Complex Network Theory [30], Graph Theory [31], and Space Syntax [11,32]. From the perspective of transportation, the current research generally has been conducted to abstract the entities (e.g., stations or regions) in a complex transportation network system as nodes and the interactions or connections (e.g., roads or railways) between entities as paths (links) to a new network, such as an undirected weighted network graph. As a result, the level of transportation network accessibility can be evaluated and characterized through the connectivity of nodes and paths [12,[33], [34], [35]]. Moreover, with the development of big data in recent years, the characterization of regional spatial accessibility and inter-regional network connections by traffic flow has received increasing attention. These studies transform the perspective of transportation network structural characteristics from “surface” to “points and lines” to characterize the regional form evolution by traffic flow. According to the performances of the intensity and tendency of the traffic flow by taking the transportation network as the carrier, the purpose is to analyze the spatial aggregation characteristics of the industry, land use, population, and their overlapping spatial economic influences on regional development [36,37].

Nevertheless, in the context of the transportation network evolution and its relationship with economic growth, many studies focus on implementing the analysis of the nodes’ spatial distribution and their location factors, and the node-link topological connections, thus explaining the adaptive correlation with the regional development in combination with the evolution of transportation network [38,39]. With the fractal geometry [40] being widely used [[41], [42], [43], [44]], some researchers have applied the spatial fractal geometry models [45] to evaluate and characterize the spatial structural characteristics of a complex transportation network system by measuring the fractal dimensions of a network, instead of building a node-link topological graph. The aim is to provide a new viewpoint for interpreting the complex and nonlinear morphological characteristics of a transportation network, thereby distinguishing the relevance and adaptability with regional economic growth [26,[46], [47], [48], [49], [50], [51], [52], [53]].

In 1967, French-American polymath Benoit Mandelbrot introduced the concept of fractal geometry and developed fractal analysis to depict objects in nature that cannot be quantified by whole-number dimensions [54,55]. Unlike Euclidean geometry (e.g., line, circle, rectangle, angle, parabola), fractal geometry evaluates a system from macro to micro level, providing quantitative spatial information by a single value, i.e., fractal dimension. The fractal dimension is a significant measurement of fractals that summarizes complexity, heterogeneity, and spatial hierarchy about an object's geometrical structure at multiple scales, realizing the capture of the spatial feature by evaluating how fast its value increases or decreases in terms of scale change [56]. The fractal dimension summarizes and expresses fractal characteristics as a number [57], and the value varies between 1 and 2 [58,59]. Fractal geometry could interpret complex natural feature formations (e.g., clouds, coastlines, plants) that show different degrees of irregularity, complexity, and self-similarity [57]. Hence, fractal geometry is an ideal choice for analyzing the dynamic urban growth and spatial organization of physical features (e.g., transportation network), providing a new way to visualize a complex system and analyze it holistically [60].

Therefore, researchers have attempted to introduce fractal geometry into the study of urban geography, including urban space management [61], urban sprawl management [62,63], urban pattern design [60], and street network [[64], [65], [66], [67]], analyze the complex organization of different physical features, thus explore its complex nature, heterogeneity, and irregularity in urban space [68]. Existing research has verified that the transportation network follows fractal properties, including urban street networks, highway networks, and railway networks [25,44,45,47,[49], [50], [51], [52],[64], [65], [66], [67]]. In the early 1990s, Benguigui first implemented research on the fractal characteristics of railway networks, revealing that the spatial structure of railway networks follows an ordinary organizational pattern [57,62]. At this point, the application of fractal dimension, e.g., the length-radius dimension, branch-radius dimension, and Hausdorff dimension (similarity dimension), in transportation network structure is increasingly expanding, and fractal dimension has gradually become an effective parameter and data support for analyzing the spatial characteristics of transportation networks including network density, topological structure, connectivity, and function, thus characterizing the spatial structure of transportation network [69,70].

A series of recent studies has found that the fractal dimension of the transportation network in a region was positively correlated with the comprehensive economic index, indicating that the more uniform the transportation network coverage, the more complex the network structure, and the better the urban land-use, economic, and environmental development of the region [[52], [53], [54],^56^,^65^,[71], [72], [73], [74]]. For instance, Lu et al. used the fractal dimension (box counting method) to characterize the correlation between the fractal dimension of urban transportation networks and urban development indicators such as population and land use. The research reveals that the fractal dimension of urban road networks increases with urban spatial expansion [71]. Sreelekha et al. attempted to explore and measure the variation of fractal dimension within a given city and compared the fractal dimension between different areas in the city. The study reflects that the minimum value corresponds to the suburban zone, while the maximum value corresponds to the central business zone [73]. The above studies have confirmed that urban development indicators such as population size and economy significantly impact the fractal characteristics of urban transportation networks [66].

Various factors influence the evolution and formation of transportation network structural characteristics, such as the geographical environment, urban planning, and socioeconomic aspects. As a result, there are significant spatial differences and heterogeneity in the morphology of the transportation network across different regions. Thus, it is worth noting that considering the impact of spatial variations is a crucial topic in the study of transportation network structural and fractal characteristics. Nevertheless, although the correlation between the fractal characteristics of transportation networks and the influencing factors has been extensively explored and analyzed, the research on the assumption of the network's spatial nonstationarity, nonlinear, and heterogeneous derived from the effects of spatial variations and socioeconomic factors still needs to strengthen. Therefore, this paper aims to employ spatial complexity, nonstationarity, and heterogeneity as a premise to explore and characterize the fractal characteristics of the high-speed railway network in China (HSRNC) and its relationship with the related influencing factors, i.e., economic growth.

In 2021, the high-speed railway network in China (HSRNC), led by the national high-speed railway trunk lines, regional passenger lines, and urban intercity railways, has become an emerging traffic mode but a significant carrier of regional transportation services. With the formation of the “Eight Vertical and Eight Horizontal” pattern of HSRNC and the continued improvement of network structure, profound changes have emerged in the regional spatial network structure and socioeconomic development. The experiences in the high-speed railway network development in developed countries or regions of the world, such as France and Japan, have demonstrated that the qualitative breakthrough in most regional development is directly related to the emergence and construction of high-speed railway network systems [75]. The high-speed railway transportation development guides the orderly urban expansion, reshapes the regional spatial structure, and promotes the connection of the regional economy, population, and land use, laying a solid foundation for supporting and guiding the coordination of regional spatial form evolution and the optimal allocation of markets and resources [76]. For example, the urban agglomeration of north-western Europe has developed a spatial pattern with Paris at its core by building a radial railway network system, including high-speed railways, metro routes, and light railways. In addition, with the integration of the high-speed railway network and land use, Tokyo has promoted the growth of the central metropolitan and the surrounding nascent small and medium-sized cities and towns, forming a multi-level and networked cluster spatial network system of cities and towns in the region. Thus, while the high-speed railway network undertakes traffic functions, the network evolution could provide more space for regional development, including regional urban expansion and spatial structure re-organization, and more impetus to regional economic growth [77,78].

Though the development of HSRNC started late compared to some developed countries, such as Japan, France, and Germany, it has been promoted rapidly in the last 20 years, even being ahead of other countries or regions in the world. Dominated by the national high-speed railway trunk lines, intercity rapid railways, and regional passenger line networks, the high-speed railway network in China meets the increasing transportation demand with the fast socio-economic development, yielding significant impacts on the transformation of regional spatial structural pattern and population mobility and the economic growth in the whole country [16,79]. In the early research on the high-speed railway network in China, the related scholars mainly discussed the spatial location impacts of the specific high-speed railway stations from the perspectives of spatial accessibility [80,81], land-use change [82,83], urban spatial expansion [84], economic coordination [26], or competition and cooperation with other transportation modes such as highways [[85], [86], [87]]. In recent years, with the rapidly increasing prominent spatial effects on the regional spatial network system of HSRNC [24,28,29], the study on the spatial distribution and structural characteristics of HSRNC and its coordinated relationship with regional development has received growing attention [85]. Therefore, this paper focuses on examining and characterizing the fractal properties and dimensions of HSRNC for evaluating and interpreting its overall spatial structural pattern in intensity, accessibility, complexity, and coverage, with the integration of the fractal theory and geography information system (GIS). The aim is to reveal the non-linear morphological characteristics and heterogeneity of HSRNC, thus exploring the coordinated relationship between network development and economic growth. This study should be favored to ensure access to the optimization of the spatial structural organization of HSRNC and the coordinated development with the regional economy and to provide a new perspective for future network planning and evolution.

## 2 Study area and methodology

### 2.1 Study area

**Fig. 1:**
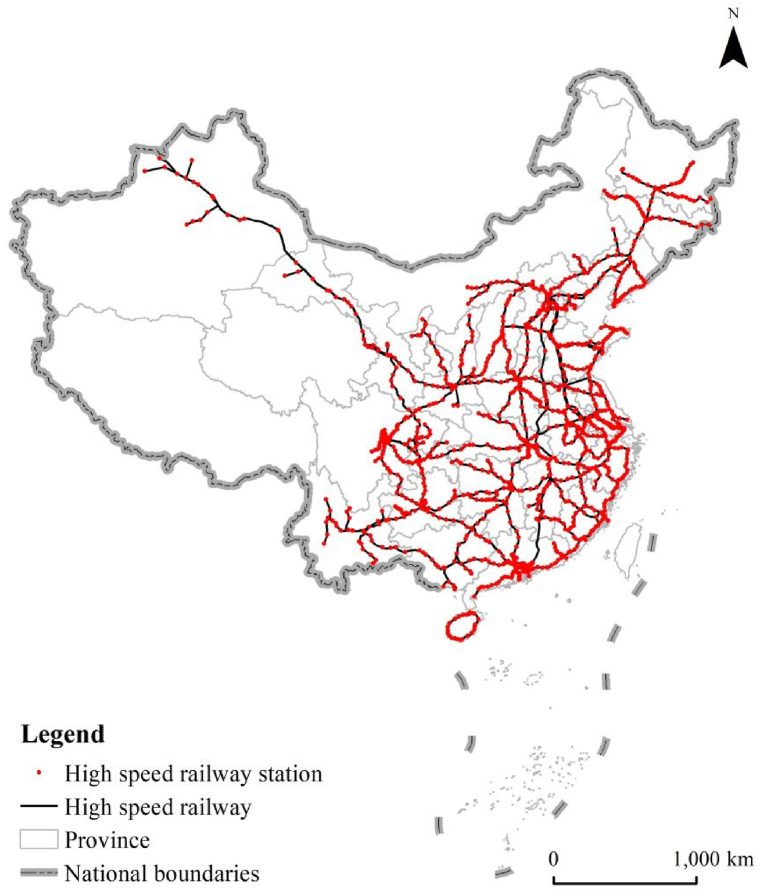
High-speed railway network in China.

In 1999 the first high-speed railway in China (i.e., Qinhuangdao-Shenyan High-speed Railway) was operated. After more than 20 years of rapid development, China built a complex high-speed railway network system with a total mileage of 37,900 km, accounting for more than two-thirds of the global mileage of high-speed railway networks. Fig. 1 shows that 31 provinces and the Hong Kong Special Administrative Region (HKSAR) in China have been covered by the high-speed railway network, including more than 15,000 km of routes (railways) with speeds up to 300 km/h. Such a complex high-speed railway network has been playing a critical role in the spatial flow of production factors, resources, and population, stimulating the regional spatial structure, economic development, and inter-regional spatial economic connections.

To further improve the coverage and service level of the high-speed railway networks, China released the medium and long-term high-speed railway network layout in 2016, which clearly proposes to build the main channels (routes) of “Eight Vertical and Eight Horizontal” to expand the original planning of “Four Vertical and Four Horizontal”. The network layout of “Eight Vertical and Eight Horizontal” covers a total mileage of 5600 km and is expected to be finished in 2026. It follows that the performance of the network planning of “Eight Vertical and Eight Horizontal” should further promote the regional spatial structure and strengthen the spatial economic connection between diffident regions such as urban agglomerations, metropolitan areas, middle-size and small cities, and towns. Furthermore, the closer combined transportation of ordinary railway and high-speed railway, as well as the national high-speed railways and the intercity railways with high-density commuter service functions, will be realized.

### 2.2 Methodology

The fractal model is built based on the fractal geometry theory, which is an effective tool for portraying the complexity and non-linear property of the observation subject by the evaluation of fractal dimensions, including length dimension, branching dimension, and similarity dimension (i.e., Hausdorff dimension). Generally, the fractal model is characterized by scale invariance, also known as self-similarity or self-affine, that is invariant under the usual geometric transformation. It requires that the structure of the occupied space should be adapted to a hyperfine structure with infinite nesting. Therefore, this study implements the fractal analysis to provide length dimension, branching dimension, and Hausdorff dimension to examine the networked and structured level in intensity, complexity, and coverage uniformity of the high-speed railway network, thus assess the network spatial accessibility, heterogeneity, nonstationarity, and variability. Furthermore, based on fractal theory, we aim to explore and characterize the coordination along with the high-speed railway network development and economic growth, particularly elucidating their non-linear correlation and adaptation through the generalized fractal dimension of non-linear systems.

#### 2.2.1 Length dimension measurement

Fractals have three fundamental characteristics: infinite repetition, self-similarity, and irregularity. Based on the extension based on a central point, i.e., the measurement center, it can generate complex and diverse fractal shapes. In fractal geometry, such a spatial extension refers to the process of repeatedly extending the surrounding shapes from a central point until the entire shape is formed. During this process, each extension will follow specific rules (e.g., a radius of gyration) to ensure the self-similarity and irregularity of the entire graph. The length dimension reflects the intensity of transportation network density distribution in an area, and the points (e.g., cities) with dense networks usually refer to the measurement centers. The peripheral range of the measurement center is calculated based on a specific radius. As a result, the length dimension of a network could be attained through the power exponential function between the regional radius and the network mileage in the region.

Specify that the length L, square S, and volume V are related to one measured M, which can describe as:

(1) $$L^{1/1}\propto S^{1/2}\propto V^{1/3}\propto M^{1/D}$$

As the transportation network within a specific square S has fractal characteristics, in terms of Equation (1), the following relationship between the total network mileage within the area L(S) and the square S can be described:

(2) $${L\left(S\right)}^{1/D}\propto S^{1/2}$$

When $S=\pi r^{2}$, then $S\propto r^{2}$. Thus Equation (2) can transform into a new expression:

(3) $$L\left(r\right)=L_{1}r^{D_{L}}$$

Where r denotes the gyration radius; L(r) identifies the total mileage of the transportation network within the area with radius r; L_1_ is a constant factor, and the power exponent D_L_ is the length dimension. By deriving Equation (3), a spatial decay equation of the density distribution of the transportation network in the area with radius r can be obtained.

(4) $$\rho \left(r\right)\propto r^{D_{L}-d}$$

Where d = 2 is the Euclidean dimension. Equation (4) shows that the different situations of spatial morphological changes (i.e., intensity changes) in the density distribution of transportation network in a region can be concluded: (1) D_L_ < 2, the network density decreases from the measured center to the periphery, which means that the overall intensity of network density in the region has not reached saturation, in other words, there is a specific potential for growth in intensity in the peripheral area; (2) D_L_ = 2, the network density varies uniformly from the measured center to its surrounding area, indicating that the network density in the region is saturated; (3) and D_L_ > 2, the transportation network density increases from the measurement center to the periphery, implying that the measurement center is not properly selected and the result is anomalous. When both D_L_ and L_1_ are at a high level, it indicates that the intensity of network spatial density tends to be saturated, and the network is more developed with better accessibility.

#### 2.2.2 Branching dimension measurement

The branching dimension is an indicator to reflect the change rate of the branching number of transportation networks. Therefore, it is applied to measure the network cross-sectional features and complex spatial variation, thus characterizing the network complexity. The branching dimension can more specifically interpret the internal transportation network structure and provide more effective support for the advanced network organization if only the network complexity is measured. Like the length dimension, the branching dimension uses the gyration radius to define the self-similar fractal properties of the branching structure of the transportation network with Equation (5).

(5) $$N\left(r\right)\propto r^{D_{B}}$$

Where r denotes the gyration radius. If a traffic hub (i.e., measurement center) in a transportation network is represented as the center of a circle, taking the gyration radius r (r = 1, 2, …, n), then it can get the n equal-width concentric circle, which gives the number k = 1, 2, …, n from the inside to the outside. Given that the sum of the branches of the transportation network in the No. k circle is N(k), which can be represented by:

(6) $$N\left(r\right)＝\sum_{k=1}^{r}N\left(k\right),k\leq r,r=1,2,\ldots ,n$$

In Equation (6), N(r) refers to the cumulative number of branches, which varies with r. When r = 1, 2, …, n, then k = 1, 2, …, r. As k ≤ r, r limits the value of k. Based on the above definition, Equation (5) is transformed into as follows.

(7) $$N\left(r\right)=N_{1}r^{D_{B}}$$

N_1_ is the constant factor, i.e., the proportionality factor, and the power exponent D_B_ is the branching dimension. Taking the logarithm of Equation (7), it can obtain

(8) $$D_{B}=\ln\left(\frac{N\left(r\right)}{N_{1}}\right)/\text{lnr}$$

In Equation (8), D_B_ refers to the branching dimension, which reflects the overall complexity of the transportation network structure in each region. The high values of N_1_ and D_B_ reflect that the transportation network is well-developed and complex. If N_1_ is high, but D_B_ is low, it presents that the network gathers around the measurement center, implying a low network development level and complexity. When N_1_ is low, but D_B_ is high, it reveals a more balanced development of the transportation network, but the transportation network may not be sound. When both values of N_1_ and D_B_ are low, it indicates the transportation network spatial distribution is uneven with poor accessibility and rudimentary network structure and organization.

#### 2.2.3 Hausdorff dimension measurement

Hausdorff dimension, i.e., similarity dimension, is a mathematical basis of fractal dimension to measure the coverage uniformity of a given transportation network and describe the network complexity and space occupation of fractal irregular graphics. Since the network coverage uniformity is higher with more uniform route distribution under the same network density, the network coverage uniformity index given by similar dimension can provide a reference for the network planning at a microscopic view, thus better meeting the requirements of the practical evaluation and planning than the traditional network density index which is suitable for the macroscopic hierarchical planning issues. Hausdorff simplified calculation method is to partition a transportation network in the given region by a square grid with side length l. The number of non-empty square lattices can count as G(l). If l changes, G(l) changes accordingly, resulting in the curve [l, G(l)]. According to the fractal theory, Equation (9) can be obtained.

(9) $$G_{i}\left(l_{i}\right)\propto l_{i}^{D},i=1,2\text{,}\ldots n,n\;is\;\text{the}\;\text{number}\;\text{of}\;\text{gradient}$$

Using the changing rate of the curve [l, G(l)] to define the similarity dimension, it has

(10) $$D\left(l\right)＝\frac{d∙\ln\;G\left(l\right)}{d∙\ln\;l}$$

If the similarity dimension is linearly distributed in the log-log plot of [l, G(l)] based on Equation (10), a fitting linear regression can obtain

(11) $$\ln\;G\left(l\right)=A-D_{G}\;\ln\;l$$

In Equation (11), D_G_ is the slope of a linear regression fitted line in the double logarithmic coordinate diagram; A refers to the intercept of the fitted line in the linear regression model. When the fractional dimension D_G_ is higher, the more routes pass through the square grid, the higher the self-similarity of the transportation network, and the more uniform the distribution and overall coverage of the network. Likewise, D_G_ < 1 indicates the poor network coverage in the region; 1≤D_G_ < 1.585 denotes the moderate level of network coverage; 1.585≤D_G_ ≤ 2 identifies the good covering of the network, especially presenting the network distribution even in some small areas [48]. Therefore, the transportation network coverage uniformity measured based on the Hausdorff dimension can reflect the overall network density distribution and explore the network coverage in each small area. From a micro perspective, the Hausdorff dimension favors characterizing the network spatial distribution form and implementing more exquisite network planning for providing practical and scientific recommendations.

#### 2.2.4 Measurement of the coordination of high-speed railway network development and economic growth

To analyze the adaptability and correlation between the high-speed railway network development and regional economic growth in China, a generalized fractal model involving the properties of high-speed railway network development and economic growth, which represents the network mileage and the regional gross domestic product (GDP), respectively, is proposed [26,52,53]. In the model, the nonlinear correlation divides the systematic fractal dimension to characterize the correspondence more accurately.

A dynamical system of transportation network development and economic growth based on the factor association is first defined [88,89]. It is simplified when only two factors are considered.

(12) $$\frac{{\text{dx}}_{i}}{\text{dt}}=a_{i}x_{i},\frac{{\text{dx}}_{j}}{\text{dt}}=a_{j}x_{j}$$

Where a_i_ and a_j_ are the relative growth coefficients, and Equation (12) is further translated into a systematic anisotropic growth equation, i.e., Equation (13):

(13) $$\frac{1}{x_{i}}\frac{dx_{i}}{\text{dt}}=b\frac{1}{x_{j}}\frac{dx_{j}}{\text{dt}}$$

Where b = a_i_/a_j_, is an anisotropic growth coefficient. By integral transformation, it can obtain

(14) $$x_{i}=\beta_{i}x_{j}^{b}$$

Where β_i_ = e^c^, is a proportionality coefficient, c represents a constant, and the anisotropic growth coefficient b refers to a scale factor. Assuming that the xi in a generalized space is Di, thus based on the geometric measure, it has

(15) $$x_{i}\propto x_{j}^{D_{i}/D_{j}}$$

Comparing Equation (14) and Equation (15), it shows that

(16) $$b=a_{i}/a_{j}=D_{i}/D_{j}$$

Therefore, Equation (16) is the dimensional expression for the heterogeneous growth of high-speed railway network and economy, and b has the generalized fractal dimensional property. By the law of anisotropic growth, too high or too low b-value predicts that one of the relevant elements tends to degenerate, and thus the systematic structure will be less diverse and stable.

The relationship between the augmenting mileage (represented by xi (i = 1, 2, …, n)) of the transportation network and the corresponding economic output, i.e., GDP denoted by y, is assumed to be

(17) $$y=\text{kf}\left(x_{1},x_{2},\ldots ,x_{n}\right)$$

In Equation (17), k is a constant. A full differential transformation gives

(18) $$y=\mu \prod x_{i}^{\sigma_{i}}$$

Where μ refers to a coefficient, and σ_i_ is the parameter that can be calculated by Equation (19).

(19) $$\sigma_{i}=\frac{\text{dlny}}{\text{dln}x_{i}}$$

Equation (18) is the general form of the Cobb-Douglas production function, which reflects the functional characteristics of transportation network development and regional economic growth based on a fractal structure from a systematic perspective. Based on Equation (14) and Equation (18), the power exponential relationship between x_i_ and y can be described as $y\propto x_{i}^{\sigma_{i}}$. If x_i_ = s and s is the total mileage of transportation network, it has

(20) $$y=as^{b}$$

Where a is a coefficient, b = σ_i,_ and σ_i_ has a generalized fractal dimension property. Given that y is D1-dimension and s represent D2-dimension, the system has fractal dimensional property if b is not an integer. When the total mileage of the high-speed railway network at a specific period (t) is L, and the regional economic output (GDP) is y, it has y = y(t). As s = L(t) is the cross-sectional data set, Equation (20) is a similar model for the elasticity of the high-speed railway network development and regional economic growth. As a result, the economic meaning of b is as follows.

B = 1 denotes that regional economic growth, i.e., regional GDP grows at the same rate as the total mileage of the regional high-speed railway network.

B > 1 denotes that the relative growing rate of regional GDP is higher than that of the total mileage of the regional high-speed railway network.

0 < b < 1 denotes that the relative growing rate of regional GDP is lower than that of the total mileage of the regional high-speed railway network.

B < 0 denotes that the relative growing rate of regional GDP decreases with the mileage augmentation of the regional high-speed railway network.

### 2.3 Data source

This paper collects the spatial data of the high-speed railway routes and stations and other geographic data (such as administrative divisions, POI (points of interest), and notes) from the Basic Geographic Database of China, which is provided by the National Geomatics Center of China (https://www.webmap.cn). The measuring scale of the collected geospatial data is 1:250,000 (China Geodetic Coordinate System 2000). A series of data processing work is implemented in the environment of ArcGIS, such as data nesting, geo-referencing, geometric rectification, and line/point identification. Meanwhile, the National Railway Construction and Planning Schematic of China (2021) is the reference to correct the data of the high-speed railways network. The economic statistics data is obtained from the socioeconomic statistical yearbooks of the provinces.

## 3 Results and discussions

### 3.1 Intensity evaluation and discussion of high-speed railway network

**Fig. 2:**
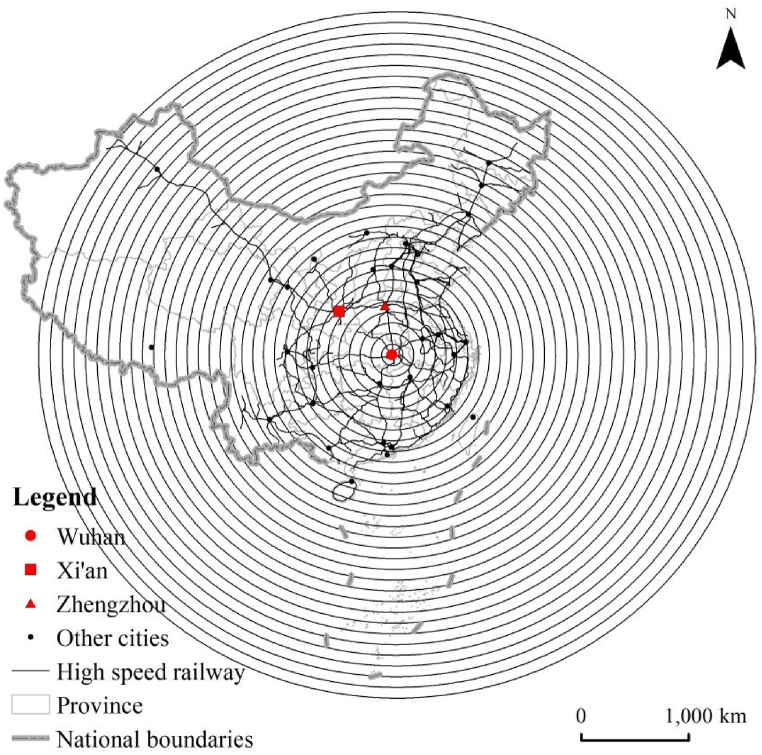
Gyration radius of high-speed railway network in China with wuhan as the measurement center.

**Fig. 3:**
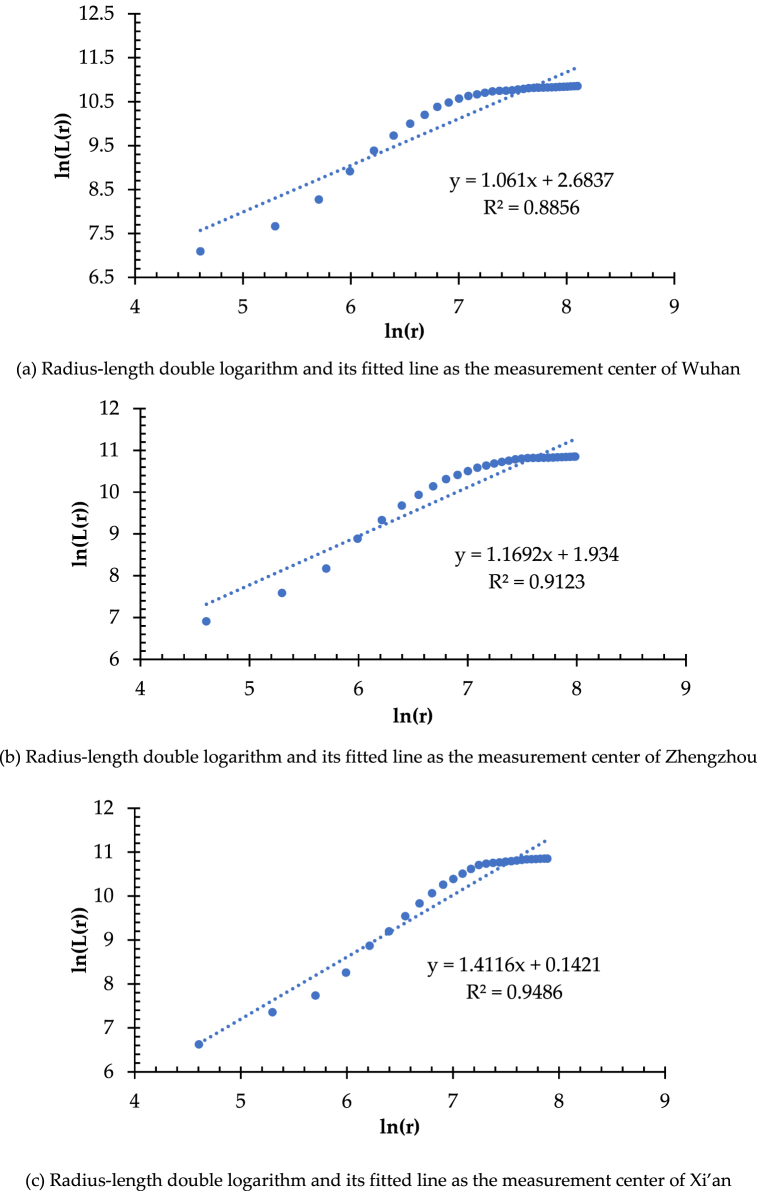
(A) Radius-length double logarithm and its fitted line as the measurement center of Wuhan. (b) Radius-length double logarithm and its fitted line as the measurement center of Zhengzhou. (c) Radius-length double logarithm and its fitted line as the measurement center of Xi'an.

In the research related to the structural organization of the high-speed railway network in China (HSRNC), the metropolises of Wuhan, Zhengzhou, and Xi'an have been explored and identified as the traffic hubs which tend to be the center of the network diagram [90]. Therefore, these three metropolises are selected as the measurement center of length dimension, respectively. As shown in Fig. 2, set the gyration radius r within 100–3200 km and the difference Δr = 100 km with Wuhan as the measurement center. R changes in Δr isotropic increments, with a total of 32 gradients. Accordingly, the GIS platform of ArcGIS performs the buffer analysis to create circular areas with Wuhan as the center and r as the radius, and a spatial intersection analysis obtains the total mileage (i.e., length) of the high-speed railway network in each circular area, which represents as L(r). As a result, the radius-length sequence of number [r, L(r)] is gotten, as shown in Fig. 3(a). If the correlation of radius (r) and length (L(r)) shows a log-linear distribution, according to the fractal theory, the high-speed railway network has fractal characteristics, and the slope of the fitted straight line refers to the value of length dimension (i.e., D_L_). Then the length dimension can be measured with Zhengzhou (Fig. 3(b)) and Xi'an (Fig. 3(c)) as the centers, respectively, in the same way, and set the gyration radius r by the different distances from the measurement center to the farthest high-speed railway station, with a uniform Δr of 100 km. The results are illustrated in Fig. 3 and Table 1, respectively.

**Table 1:** Length dimension of high-speed railway network.

Measurement Centers	r\km	Δr\km	Gradient\No.	D_L_	R^2^
Wuhan	100–3200	100	32	1.0610	0.8856
Zhengzhou	100–3000	100	30	1.1692	0.9123
Xi'an	100–2700	100	27	1.4116	0.9486

As shown in Table 1, for the high-speed railway network in China, the values of D_L_ are different with diverse measurement centers and range of gyration radius. Moreover, all the values of D_L_ for the three measurement centers (i.e., Wuhan, Xi'an, and Zhengzhou) are less than 2, which means that the spatial density distribution in the intensity of HSRNC decreases from each measurement center to its periphery. It also confirms the correct choice of measurement centers to calculate the length dimension. Moreover, Table 1 illustrates that both fits of the radius-length double logarithm with Wuhan and Zhengzhou as the measurement centers are worse than that of Xi'an, but their correlation coefficients R^2^, which are 0.8856 and 0.9123, respectively, indeed reveal the significant linear relationship (see Fig. 3(a) and (b)). Regression analysis of the three length-radius double logarithmic series derived from the measurement centers of Xi'an, Zhengzhou, and Wuhan using Least Squares shows that all are significant at the level of p < 0.0001, reflecting the apparent spatial fractal characteristics of HSRNC. It is worth noting that the radius-length double logarithm with Xi'an as the measurement center demonstrates the best fit (R^2^ = 0.9486) and the highest value of length dimension (D_L_ = 1.4116), implying that the high-speed railway network mileage grows fastest and the network density distribution in intensity decreases slowest when gradually expanding to the surrounding areas from Xi'an (see Fig. 3(c)). Comparatively, with the measurement center of Wuhan, the growing trend of the network mileage is the most moderate, and the network density distribution in intensity reduces at the fastest rate. The above results show that Wuhan has the most densely distributed high-speed railway network in its surrounding areas, followed by Zhengzhou, and Xi'an is the last.

**Fig. 4:**
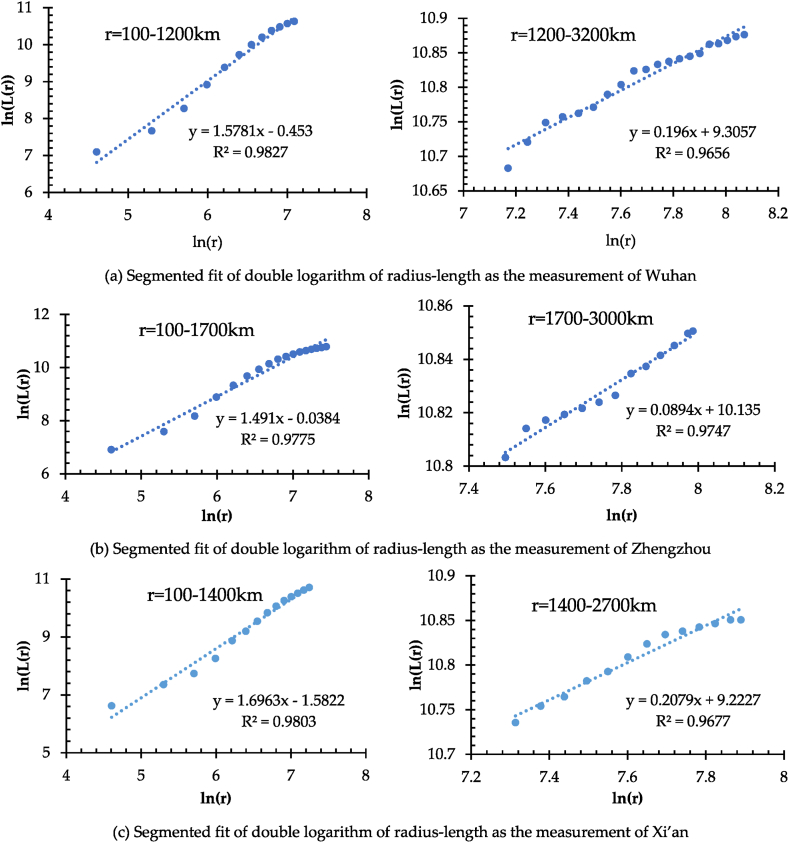
(a) Segmented fit of double logarithm of radius-length as the measurement of Wuhan. (b) Segmented fit of double logarithm of radius-length as the measurement of Zhengzhou. (c) Segmented fit of double logarithm of radius-length as the measurement of Xi'an.

In addition, Fig. 3 presents that the scatter plots and fitted line plots of radius-length double logarithms for the measurement centers of Xi'an, Zhengzhou, and Wuhan have evident segmentation characteristics, respectively. The spatial dividing point appears at the 12th radius of the gyration gradient, i.e., r = 1200 km for Wuhan, r = 1700 km for Zhengzhou, and r = 1400 km for Xi'an. Therefore, the scatter plots and fitted lines of radius-length double logarithms for each measurement center are fitted by two segments, respectively (Fig. 4(a) and (b), and Fig. 4(c)). As a result, the correlation coefficient R^2^ of the two fitted lines is higher than 0.96, showing a better double logarithm linear fit. Nevertheless, the slope of the fitted lines (i.e., the values of D_L_) derived from the two segments shows an apparent difference. It indicates that the network density distribution in the intensity of the internal and external regions is different, marked by the dividing point.

**Fig. 5:**
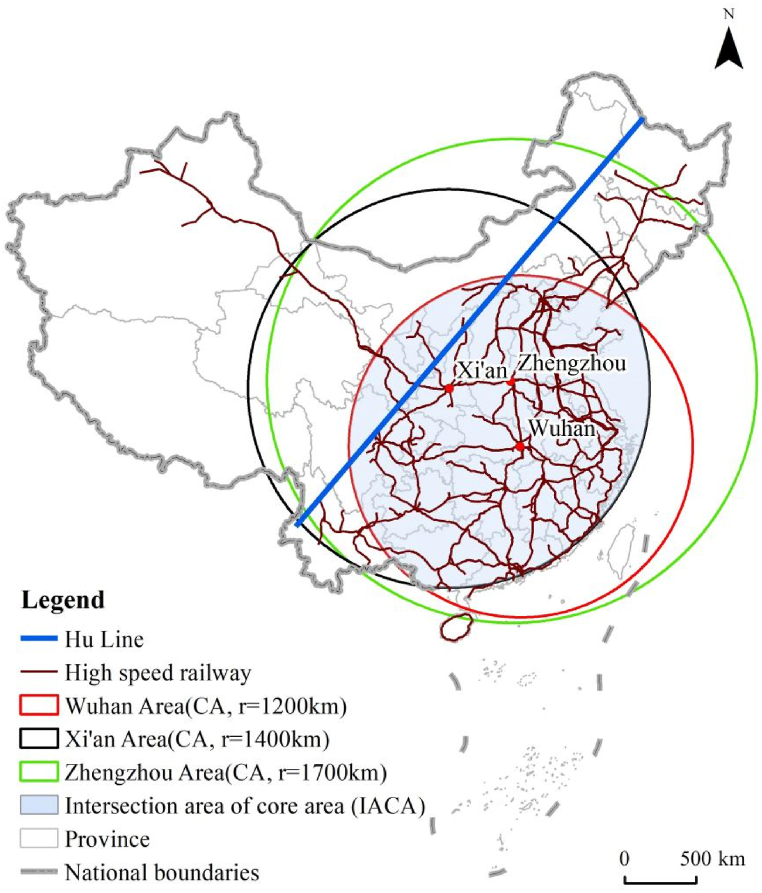
Core areas of high-speed railway network density distribution.

To further discuss the spatial intensity and heterogeneity of HSRNC, ArcGIS is applied to delimit the three circular areas denoted as the core areas (CA) with Wuhan, Zhengzhou, and Xi'an as the measurement centers based on the gyration radius of the spatial dividing points, i.e., 1200 km, 1700 km, and 1400 km, respectively. Accordingly, these three core areas are named Wuhan Area, Zhengzhou Area, and Xi'an Area, respectively, as shown in Fig. 5. Table 2 shows that the values of D_L_ in these three core areas are 1.5781 (Fig. 4(a)), 1.4910 (Fig. 4(b)), and 1.6963 (Fig. 4(c)), respectively, which are close to or even reach the interval of 1.6–1.8. However, the values of D_L_ in the regions outside the core areas are 0.1960 (Fig. 4(a)), 0.0894 (Fig. 4(b)), and 0.2079 (Fig. 4(c)), respectively, revealing the significant spatial differences between the core areas and their peripheral areas. It follows that the network density distributions within Wuhan Area, Zhengzhou Area, and Xi'an Area show a better intensity and higher accessibility level. However, in their peripheral areas, the accessibility level is significantly weaker and lower. It illustrates the spatial unevenness and heterogeneity in the intensity of HSRNC.

**Table 2:** Segment fit of length dimension and correlation coefficients.

Measurement Centers	Radius of CA	D_L_ of CA	R^2^ of CA	D_L_ of Non-CA	R^2^ of Non-CA
Wuhan	1200 km	1.5781	0.9827	0.1960	0.9656
Zhengzhou	1700 km	1.4910	0.9775	0.0894	0.9747
Xi'an	1400 km	1.6963	0.9803	0.2079	0.9677

The core areas of Wuhan Area, Zhengzhou Area, and Xi'an Area have a common intersection area, i.e., the intersection area of core areas (IACA). As shown in Fig. 5, the IACA is in the southeast of Hu Line and covers the most economically developed regions in China with a dense population, including four municipalities, i.e., Beijing, Shanghai, Chongqing, and Tianjin, as well as eastern coastal economically developed provinces (Guangdong, Fujian, Zhejiang, Jiangsu, and Shandong) and the populous provinces in the central and western regions (Henan, Hubei, Hunan, Anhui, Jiangxi, Shanxi, Sichuan, Shaanxi, Ningxia, and Guangxi). From the perspective of regional spatial structure and land use, the IACA is composed of the most maturely developed metropolitan areas and urban agglomerations in China, including the six major urban agglomerations, i.e., Beijing-Tianjin-Hebei, Yangtze River Delta, Guangdong-Hong Kong-Macao Greater Bay Area, Middle Yangtze River, Chengdu-Chongqing and Shandong Peninsula. These six major urban agglomerations have the most rapid economic development and the largest population in China. As a result, the GDP in the IACA has exceeded 10 billion by 2021, accounting for about 88 % of the total GDP of China, and the population has been close to 1.1 billion, 78 % of the total population of China. Thus, the spatial density distribution in the intensity of the high-speed railway network in China is highly compatible with the regional spatial structure, land use, and socioeconomic development.

### 3.2 Complexity evaluation and discussion of the high-speed railway network

The measurement of length dimension verifies that Wuhan, Zhengzhou, and Xi'an are suitable for measurement centers. It reveals the distinct spatial fractal characteristics in the network density in intensity, thus discovering significant spatial heterogeneity, especially between the core areas and their surrounding areas. Therefore, this study further explores the branching dimension for characterizing the spatial structural complexity of HSRNC, specifically in the IACA. Wuhan, which is in the geometric center of the IACA and has a dense distribution of high-speed railway network in its surrounding area, is determined as the measurement center of the evaluation of the branching dimension.

**Fig. 6:**
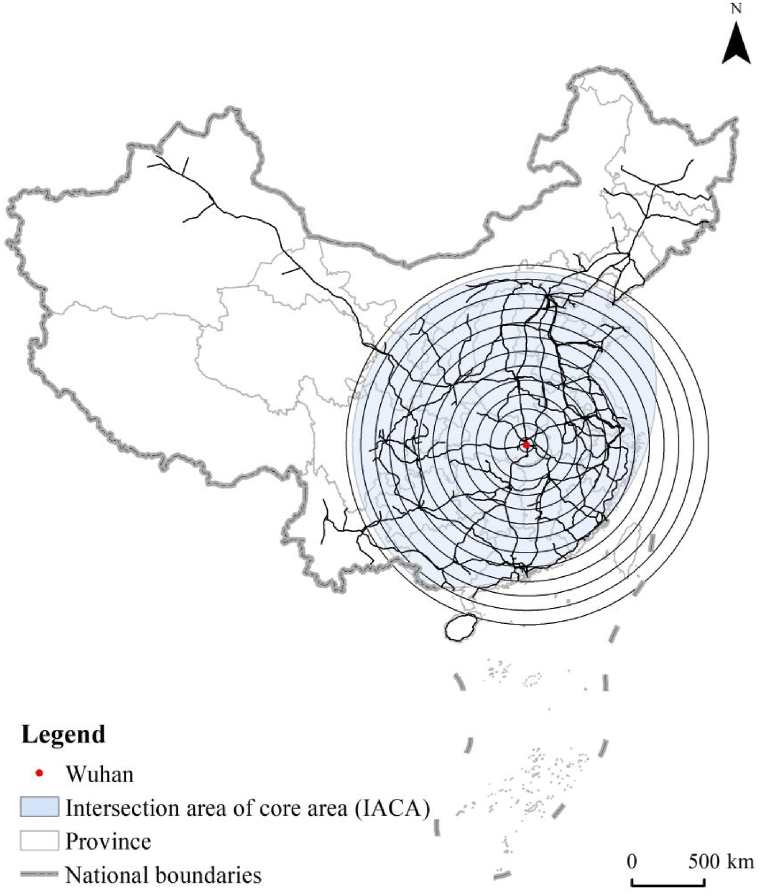
Gyration radius of the IACA.

**Fig. 7:**
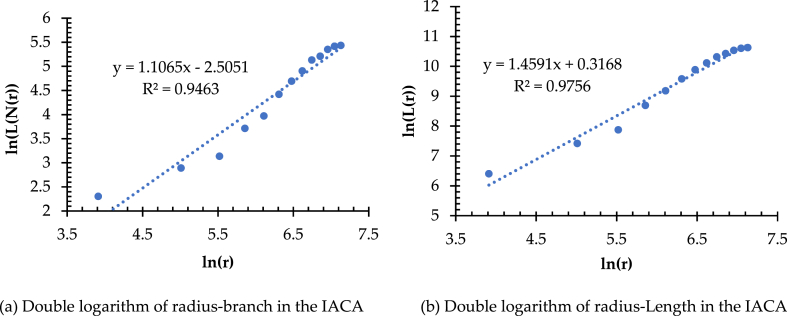
(A) Double logarithm of branch-radius in the IACA. (b) Double logarithm of length-radius in the IACA.

**Fig. 8:**
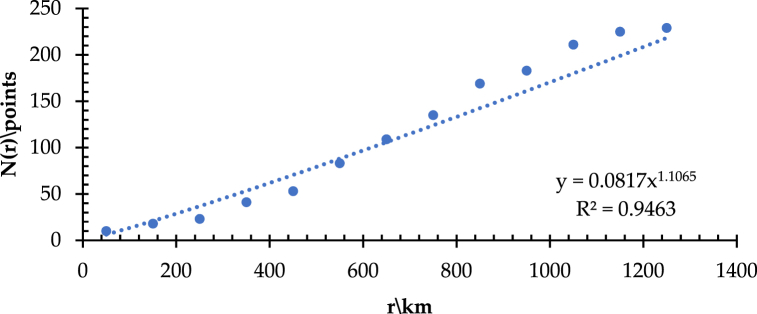
Scatter plot and multiplicative power trend line of radius-branch in the IACA.

The branching dimension represented by D_B_ is the significant index to evaluate the spatial complexity of a transportation network. The value of D_B_ presents that the faster the total number of network bifurcations increases with the increasing radius from the measurement center to the surrounding area. If the value of D_B_ is high, it means that the network structure is complex, the network coverage capacity is strong, and the connectivity/accessibility is good. Conversely, it indicates a simple network structure and poor accessibility. Like the method applied in the measurement and analysis of length dimension, D_B_ is measured in the IACA and Wuhan as the measurement center, taking the gyration radius r from the range of 50–1250 km with equal increments of Δr = 100 km to form a total of 13 gradients (Fig. 6). The spatial analysis provided by ArcGIS obtains both the series of radius-length and radius-branching, which refer to [r, L(r)] and [r, N(r)], respectively. Thus, Fig. 7 shows that the correlation coefficients (R^2^) of the fitted lines of [r, L(r)] and [r, N(r)] are 0.9463 (Fig. 7(a)) and 0.9756 (Fig. 7(b)), respectively, which are well-fitted. The regression analysis tested by the least squares indicates that both the significance levels are at a = 0.0001, reflecting the significant fractal characteristics of the high-speed railway network in the IACA. It is worth noting that the value of D_B_ is 1.1061. It means that the total bifurcations of the high-speed railway network in the IACA grow relatively smoothly, forming a complex network structure with a certain penetration and service capability. However, the value of D_L_ is 1.4591. It implies that the value of D_B_ is smaller than that of D_L_, i.e., the network density in spatial intensity develops more than its structural complexity in the IACA. Therefore, there is still much potential to improve the network complexity in the IACA. As shown in Fig. 8, the scatter plot of the radius-branching series [r, N(r)] obtains a multiplicative power trend line, and the results show N_1_ as 0.0817. The lower value of N_1_ and higher value of DB reflect the relatively balanced development of the high-speed railway network in the IACA, but not yet sound.

**Fig. 9:**
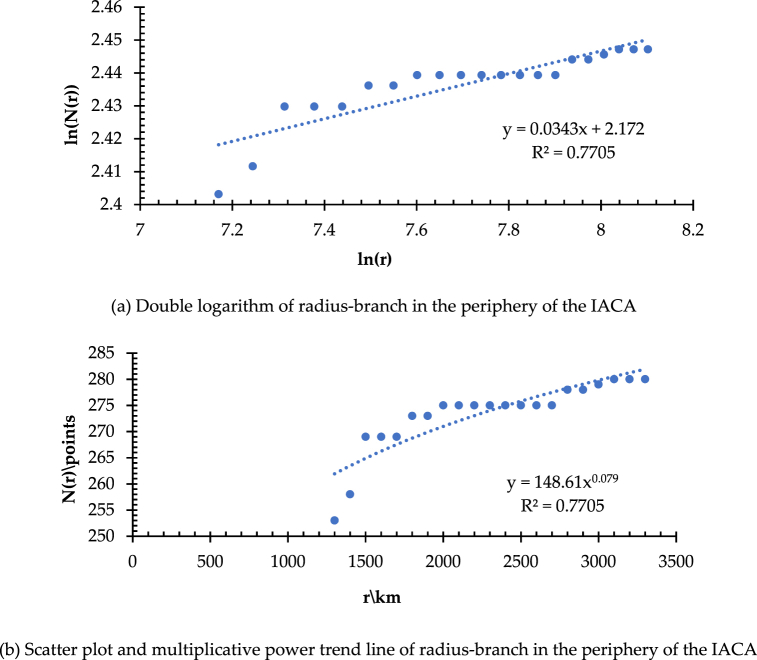
(a) Double logarithm of the radius-branch in the periphery of the IACA. (b) Scatter plot and multiplicative power trend line of radius-branch in the periphery of the IACA.

Meanwhile, to further discuss the spatial heterogeneity in the complexity of HSRNC in the context of the fractal characteristics of the branching dimension, the gyration radius r is set at 1250–3300 km (Δr = 100 km) with Wuhan as the measurement center to examine the value of D_B_ in the surrounding area of the IACA. As shown in Fig. 9(a), the number of branches in the network augments slowly in the peripheral region compared with that in the IACA as the gyration radius increases. The slope of the fitted line (D_B_) is 0.0343. According to Fig. 9(b), the higher N_1_ of 148.61, quite different from that derived from the IACA, indicates that the network structural complexity and accessibility in the peripheral area of the IACA are at a low level, and verifies that there is a significant spatial inhomogeneity in the complexity of HSRNC.

### 3.3 Coverage uniformity evaluation and discussion of the high-speed railway network

**Fig. 10:**
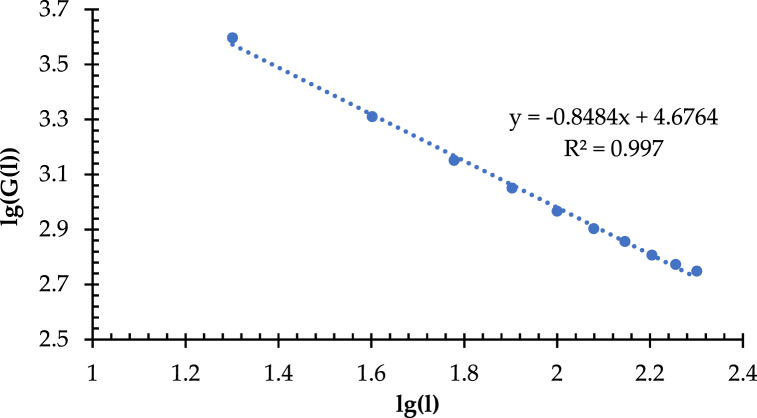
Double logarithm and fitted line of length-square lattice quantity.

Hausdorff dimension (D_G_), known as similarity dimension, is the mathematical basis of fractal dimension, which aims to describe the network complexity and space occupation (i.e., coverage), revealing the difference in the coverage uniformity of traffic route distribution under the same transportation network density. Compared with the traditional transportation network density calculation, i.e., ratio of mileage to area, the Hausdorff dimension can meet the requirements for evaluating and characterizing the network coverage uniformity and complexity from a micro point of view. The simplified calculation method of the Hausdorff dimension generally uses a regular square grid with a specific side length (l_i_). It partitions the network service coverage area, thus counting the intersections between the routes and the square lattice, denoting G (l_i_). G (l_i_) varies with the changes of l_i_, forming a length-square lattice quantity series, i.e., [l, G(l)]. It can further plot in a double logarithmic coordinate plot and a fitted line plot. As shown in Fig. 10, the regular square lattices with side lengths (l) ranging from 20 to 200 km are determined, with a total of 10 gradients from their side lengths are increased by Δl (20 km) in equal increments, and thus obtaining the length-square lattice quantity series [l, G(l)] of the high-speed railway network in China.

Fig. 10 illustrates that R^2^, the correlation coefficient for the double logarithmic fitted line of l and G(l), is 0.997, which is significant at the level of p < 0.0001 in the regression analysis gained from using least squares. It implies a high fitting accuracy and further verifies the distinct fractal characteristics of HSRNC. However, it found that the value of D_G_ is 0.8484, which is lower than 1.0, demonstrating the lower level of network coverage uniformity in the nationwide area.

**Fig. 11:**
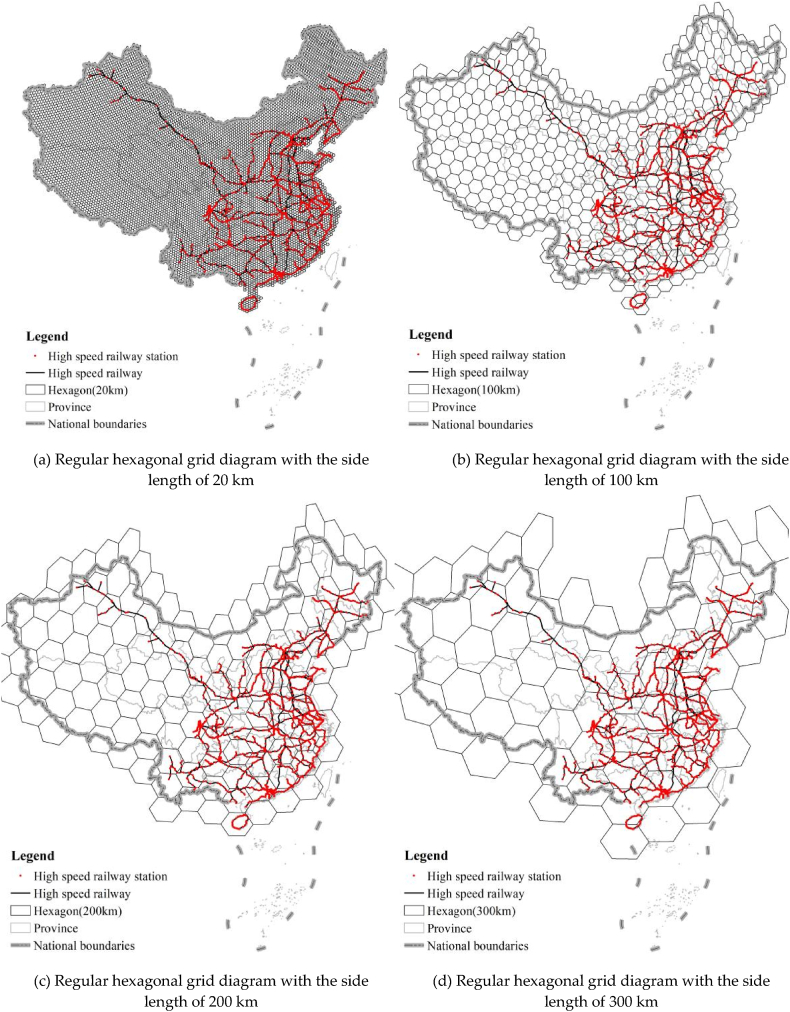
(a) Regular hexagonal grid diagram with the side length of 20 km. (b) Regular hexagonal grid diagram with the side length of 100 km. (c) Regular hexagonal grid diagram with the side length of 200 km. (d) Regular hexagonal grid diagram with the side length of 300 km.

**Fig. 12:**
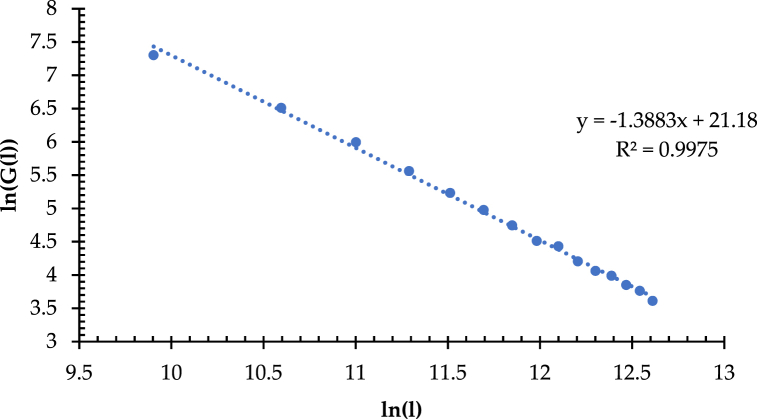
Double logarithm and fitted line of the length-positive hexagonal grid quantity.

The Central Place Theory [88,89] assumes that if an area has a honeycomb structure, which forms under a specific radius, the services provided by the region are the largest. Therefore, in this study, the hexagonal locality model is used to re-measure the Hausdorff dimension to explore an accurate interpretation of the structural complexity and space occupation (coverage) of HSRNC. As shown in Fig. 11, the regular square lattice mentioned before forms by the hexagonal honeycomb structured grid with side lengths (l) ranging from 20 to 300 km, whose side lengths increase in equal increments by Δl = 20 km. It obtains a total of 15 gradients. Fig. 11(a), (b), Fig. 11(c), and Fig. 11(d) show the regular hexagonal grid diagrams with the side lengths (l) of 20 km, 100 km, 200 km, and 300 km, respectively. As a result, Fig. 12 illustrates the scatter plot of the length-positive hexagonal grid quantity series [l, G(l)].

**Fig. 13:**
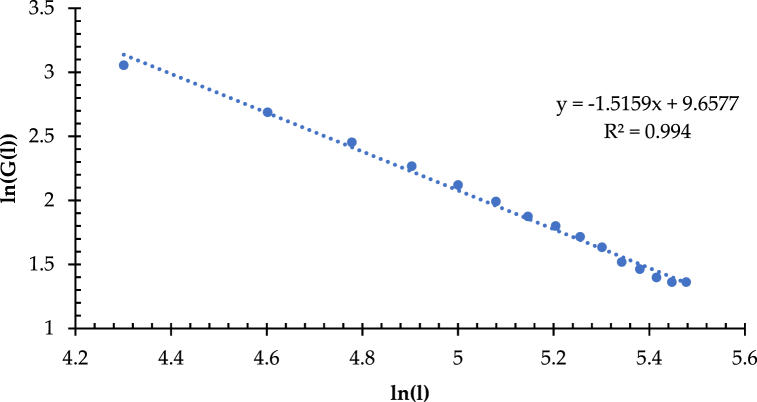
Double logarithm and fitted line of length-regular hexagonal grid quantity.

Fig. 12 shows that the correlation coefficient (R^2^) is 0.9975, which is significant at the level of p < 0.0001, with slightly higher fitting accuracy than the length-regular square quantity. Note that the value of D_G_ is 1.3883, which is more than 1. It reflects that the overall coverage uniformity of the high-speed railway network in China is at a moderate level, which is more in line with the current network development. The same method measures the branching dimension (D_B_) in the region of IACA for discussing the spatial differences in the network coverage uniformity and that in the nationwide area. The correlation coefficient (R^2^) of the linear fit of the double logarithm in the region of IACA is 0.994, which is significant at a = 0.0001. As shown in Fig. 13, the value of D_B_ is 1.5159 in the IACA, which is significantly higher than that in the nationwide area. It implies that in the IACA, the network coverage is even in the small plots. Thus, the network structural complexity and coverage uniformity in the IACA is better than that in the nationwide area in the context of a micro perspective.

### 3.4 Fractal characteristics of the high-speed railway network in the urban agglomerations

**Fig. 14:**
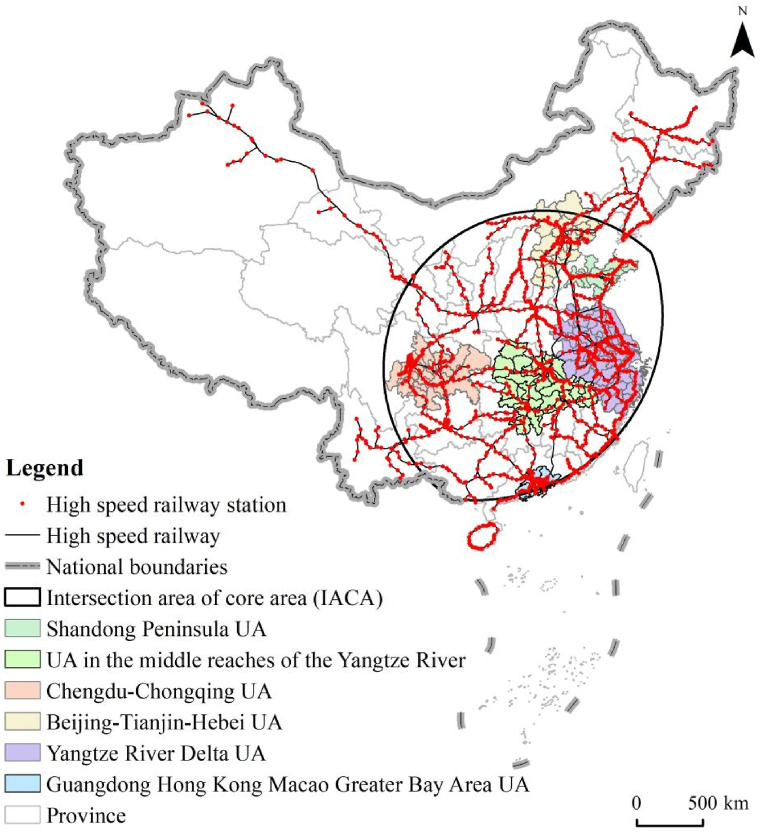
Urban agglomerations in the IACA.

This paper further analyzes the fractal dimensions (i.e., length dimension, branching dimension, and Hausdorff dimension) of the high-speed railway network in the six urban agglomerations (UA), including Beijing-Tianjin-Hebei, Yangtze River Delta, Guangdong-Hong Kong-Macao Greater Bay Area, Middle Yangtze River, and Shandong Peninsula, which are in the IACA (Fig. 14). The aim is to explore the fractal characteristics of the high-speed railway network and the spatial differences in the network intensity, accessibility, and complexity. According to the measurement of D_L_ and D_B_, firstly, the hub station of the high-speed railway network within each urban agglomeration is identified as the measurement center, which is Beijing South Station, Shanghai Hongqiao Station, Guangzhou South Station, Chongqing West Station, and Wuhan Station and the Jinan Station, repressively; secondly, the gyration radius r is in line with the regional scope of each urban agglomeration, including 20–450 km for Beijing-Tianjin-Hebei and Yangtze River Delta, 20–190 km for Guangdong-Hong Kong-Macao Greater Bay Area, 20–340 km for Chengdu-Chongqing, 20–530 km for Middle Yangtze River, and 20–490 km for Shandong Peninsula, and Δr is 10 km for each UA (Table 3). The values of D_L_ of the urban agglomerations shown in Table 3 range from 0.9 to 1.2. All the values are less than 2.0. It indicates that the high-speed railway network density in intensity in each UA decreases from the measurement center to the periphery. Also, it reflects the appropriate determination of the measurement centers in urban agglomerations. It follows that the intensity of the network density in the peripheral area requires further enhancement. It is worth noting that the values of D_L_ and L_1_ shown in Table 3 are both at a medium level, implying that the high-speed railway network density in intensity in each UA has not reached saturation. As illustrated in Table 3, D_B_ and N_1_ are at a lower level, reflecting that the network structure is relatively simple and the accessibility level is low in each UA. Accordingly, Table 3 shows that the values of D_L_ of all urban agglomerations are higher than those of D_B_. It reveals that the network density in intensity within each UA is better than the network complexity. Thus, the six major urban should agglomeration strengthen the network's structural organization and spatial accessibility.

**Table 3:** Length dimension and branching dimension of the high-speed railway network in the urban agglomerations.

UA	r\km	Δr \km	Length Dimension			Branching Dimension		
			D_L_	R^2^	L_1_	D_B_	R^2^	N_1_
Beijing-Tianjin-Hebei	20–450	10	0.9365	0.9708	9.0903	0.7133	0.9375	0.8252
Yangtze River Delta	20–450	10	1.1765	0.9852	4.5149	0.7829	0.9367	0.4608
Guangdong-Hong Kong-Macao Greater Bay Area	20–190	10	0.9820	0.9613	11.935	0.7435	0.8927	0.5168
Chengdu-Chongqing	20–340	10	1.1238	0.9965	9.4959	0.7882	0.8878	0.2002
Middle Yangtze River	20–530	10	0.9326	0.9774	15.990	0.4908	0.8785	1.3853
Shandong Peninsula	20–490	10	0.8977	0.9908	12.956	0.6830	0.8351	0.2360

When measuring the Hausdorff dimension, i.e., D_G_, the hexagonal side lengths in each UA are set to 10–150 km with Δl = 10 km. As shown in Table 4, the linear fit of the double logarithm of the length-regular hexagonal grid quantity is superior and significant at the level of a = 0.0001. As a result, all the values of D_G_ are within the interval of [1,1.585], indicating that the coverage uniformity of the high-speed railway networks in the urban agglomerations is all at a moderate level, and relatively close but slightly different, with the urban agglomeration of Yangtze River Delta having the highest level of coverage uniformity and Guangdong-Hong Kong-Macao Greater Bay Area the lowest level of coverage uniformity.

**Table 4:** Hausdorff dimensions of high-speed railway networks in urban agglomerations.

UA	l\km	Δl \km	D_G_	R^2^
Beijing-Tianjin-Hebei	10–150	10	1.2925	0.9967
Yangtze River Delta	10–150	10	1.3543	0.9925
Guangdong-Hong Kong-Macao Greater Bay Area	10–150	10	1.1719	0.9776
Chengdu-Chongqing	10–150	10	1.2120	0.9839
Middle Yangtze River	10–150	10	1.2545	0.9932
Shandong Peninsula	10–150	10	1.2170	0.9852

### 3.5 Evaluation of the coordination between high-speed railway development and economic growth

**Fig. 15:**
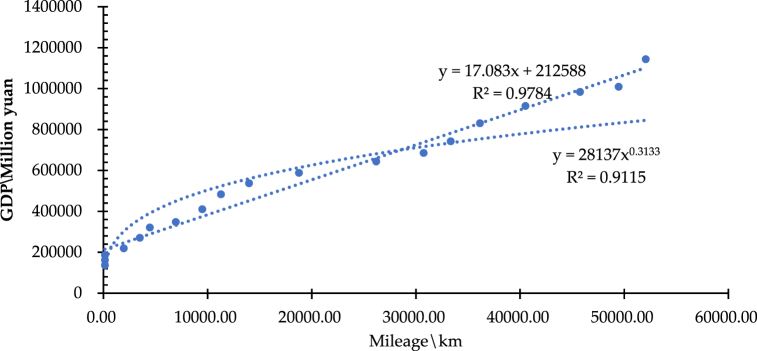
Fitted line and multiplicative power index for network mileage-GDP.

**Fig. 16:**
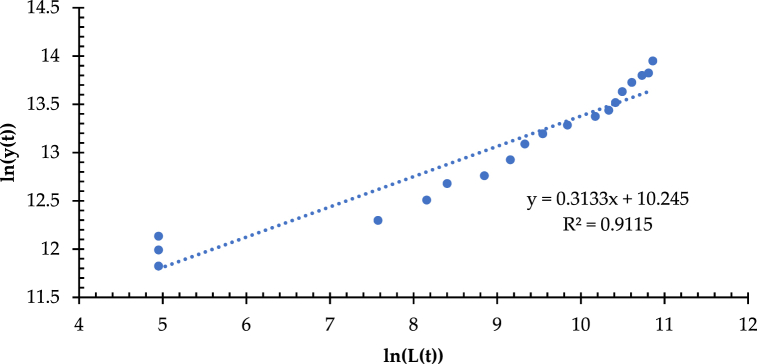
Double logarithmic scatter plot and fitted line for network mileage-GDP.

Table 5 gives the data on the total mileage of HSRCN and the gross domestic product (GDP) from 2003 to 2021. Based on the data, Fig. 15 demonstrates a distinct linear relationship between the mileage (identified as the variable of y) of the high-speed railway network in China (HSRCN) and the economic output (GDP) (denoted as the variable of s), with a correlation coefficient (R^2^ = 0.9784). It illustrates that the fractal characteristics of the coordination between y and s, i.e., the power exponential relationship model presented by Equation (20), is also a better fit with a correlation coefficient (R^2^ = 0.9136). Equation (20) can be reduced to y(t) = aL(t)b by taking the network mileage s (i.e., L(t)) and the economic output (GDP) (i.e., y(t)). Then, taking logarithms of both sides simultaneously, it has ln (y(t)) = ln(a) + bln (L(t)) (see Fig. 16). Therefore, the regression analysis implements the calculation of the corresponding ln (y(t)) and ln (L(t)), which is then performed on ln (y(t)) and ln (L(t)) using the method of least squares to determine whether the linear correlation is evident.

**Table 5:** High-speed railway network mileage and economic output (GDP) in China from 2003 to 2021.

Year	Network Mileage\km	Economic Output (GDP)\0.1 b Yuan
2003	2003.00	136,576
2004	4007.00	161,415
2005	6012.00	185,999
2006	8018.00	219,029
2007	10025.00	270,704
2008	12033.00	321,230
2009	14042.00	347,935
2010	16052.00	410,354
2011	18063.00	483,393
2012	20075.00	537,329
2013	22088.00	588,141
2014	24102.00	643,563
2015	26117.00	685,571
2016	28133.00	742,694
2017	30150.00	830,946
2018	32168.00	915,244
2019	34187.00	983,751
2020	36207.00	1,008,783
2021	38228.00	1,143,670

**Fig. 17:**
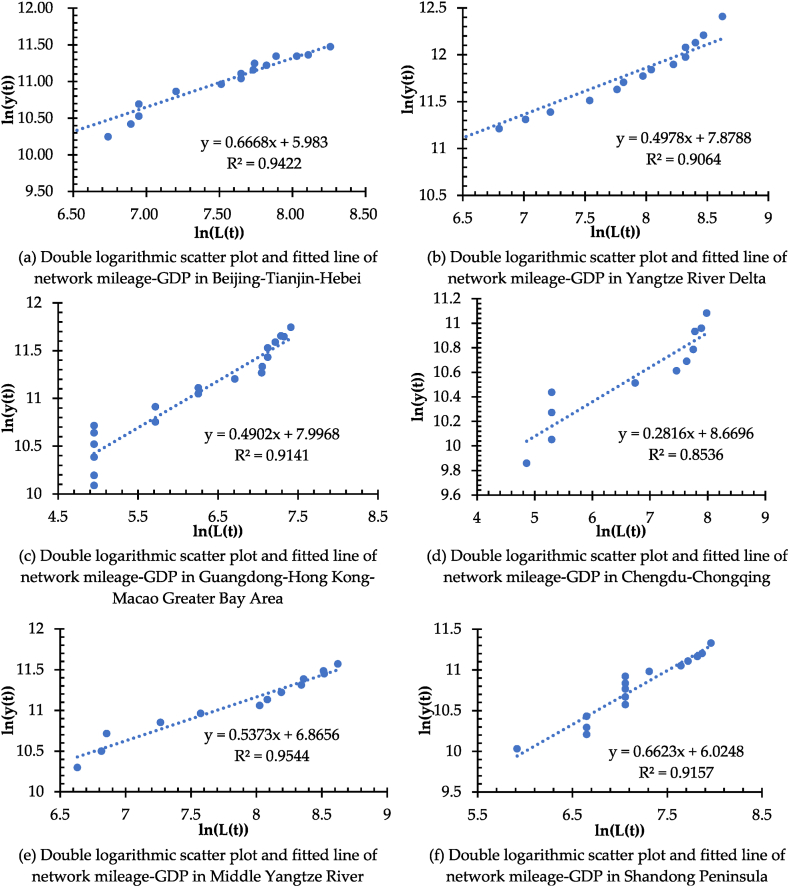
(a) Double logarithmic scatter plot and fitted line of network mileage-GDP in Beijing-Tianjin-Hebei. (b) Double logarithmic scatter plot and fitted line of network mileage-GDP in Yangtze River Delta. (c) Double logarithmic scatter plot and fitted line of network mileage-GDP in Guangdong-Hong Kong-Macao Greater Bay Area. (d) Double logarithmic scatter plot and fitted line of network mileage-GDP in Chengdu-Chongqing. (e) Double logarithmic scatter plot and fitted line of network mileage-GDP in Middle Yangtze River. (f) Double logarithmic scatter plot and fitted line of network mileage-GDP in Shandong Peninsula.

Likewise, the fractal model is applied to measure the coordinated relationship of network development and economic growth in the six major urban agglomerations of Beijing-Tianjin-Hebei, Yangtze River Delta, Guangdong-Hong Kong-Macao Greater Bay Area, Chengdu-Chongqing, Middle Yangtze River, and Shandong Peninsula from 2003 to 2021, respectively. In Fig. 17, the scatter plots of ln (y(t)), ln (L(t)), and fitted line plots of each urban agglomerations is illustrated, respectively. It shows a double logarithmic dynamic similarity between the network development and the regional economic growth for the urban agglomerations, including Beijing-Tianjin-Hebei (Fig. 17(a)), Yangtze River Delta (Fig. 17(b)), Guangdong-Hong Kong-Macao Greater Bay Area (Fig. 17(c)), Chengdu-Chongqing (Fig. 17(d)), Middle Yangtze River (Fig. 17(e)), and Shandong Peninsula (Fig. 17(f)). It provides a way to access and examine the data from time series (from 2003 to 2021) of y(t) (represented by the GDP) and L(t) (represented by the network mileage) for the whole country and the six major urban agglomerations.

Thus, Equation (20) calculates the dynamic similarity coefficient (b) (Table 6). It aims at exploring and characterizing the spatiotemporal differences and heterogeneity between the regions in the context of the coordinated relationship between network development and economic growth. Table 6 indicates that the GDP growth and high-speed railway network mileage increase in each region have a strong correlation. Moreover, the generalized fractal dimension of coordination is not an integer, which implies that the coordinated relationship between economic growth and network development has a generalized fractal dimension property as defined in Equation (20).

**Table 6:** Generalized fractal dimension in nationwide area and urban agglomerations.

Region/UA	b	R^2^	Regression Analysis of ln (y(t)) and ln (L(t))
			*P*-value
Nationwide area	0.3133	0.9115	2.22E-10 < 0.0001
Beijing-Tianjin-Hebei	0.6668	0.9422	4.61E-10 < 0.0001
Yangtze River Delta	0.4978	0.9064	4.64E-08 < 0.0001
Guangdong-Hong Kong-Macao Greater Bay Area	0.4902	0.9141	1.73E-10 < 0.0001
Chengdu-Chongqing	0.2816	0.8536	4.85E-05 < 0.0001
Middle Yangtze River	0.5373	0.9544	1.01E-08 < 0.0001
Shandong Peninsula	0.6623	0.9157	2.35E-08 < 0.0001

As shown in Table 6, the anisotropic growth coefficient of high-speed railway network development and economic growth is 0 < b < 1, which shows that the high-speed railway network develops relatively faster than the economic growth. Among them, the generalized fractal dimension b = 0.3133 in the nationwide area indicates that the growth rate of network mileage is much faster than that of GDP, i.e., economic growth, in the past 20 years. Also, the generalized fractal dimensions of 0.6668, 0.6623, and 0.5373 for the urban agglomerations of Beijing-Tianjin-Hebei (Fig. 17(a)), Shandong Peninsula (Fig. 17(f)), and Middle Yangtze River (Fig. 17(e)), respectively, are relatively higher, reflecting a better mutual adaptation of the network development and economic growth in these three urban agglomerations, where have set up the developed railway networks before the 21st century. Meanwhile, the generalized fractal dimension of the urban agglomeration of Chengdu-Chongqing is the lowest at 0.2816 (Fig. 17(d)). The reason is that in the early period, the economic growth and railway infrastructure development of Chendu-Chongqing were inferior to other urban agglomerations. Since the first high-speed railway trunk line in the urban agglomeration of Chengdu-Chongqing was in operation in 2009, it marks the epoch of rapid development of the high-speed railway network system. As a result, in the past 20 years, compared with the economic growth, the high-speed railway network development in the urban agglomeration of Chengdu-Chongqing has increased rapidly. Last, the generalized fractal dimension of both the urban agglomerations of the Yangtze River Delta (Fig. 17(b)) and the Guangdong-Hong Kong-Macao Bay Area (Fig. 17(c)) is close to 0.5, stressing the point that the growth rate of high-speed railway network mileage significantly outpaces that of economic output (GDP) in these two regions. The main reason is that they are the most developed metropolitan areas with the maximum transportation demand and the highest population mobility in China over the past two decades, which have driven the rapid development of their high-speed railway networks.

Further observation derived from Fig. 16, Fig. 17 reveals that both scatter plots show apparent segmentation. Therefore, it redraws the scatter plots and fitted lines of ln (y(t)) and ln (L(t)) for the nationwide areas and urban agglomerations by two segments, respectively, to explore and characterize the spatial-temporal heterogeneity of the coordination of high-speed railway network development and regional economic growth, as shown in Table 7.

**Table 7:** Results of generalized fractal dimensional segmentation measurement.

Region	Time Demarcations	First Half Period		Last Half Period	
		b	R^2^	b	R^2^
Nationwide area	2007	0.1475	0.8016	0.5019	0.9684
Beijing-Tianjin-Hebei	2010	1.6872	0.8045	0.6180	0.9296
Yangtze River Delta	2013	0.3512	0.9250	1.0154	0.9243
Guangdong-Hong Kong-Macao Greater Bay Area	2012	0.4764	0.7561	1.1871	0.9000
Chengdu-Chongqing	2013	0.2770	0.5410	0.9234	0.9267
Middle Yangtze River	2014	0.4994	0.8436	0.7925	0.9795
Shandong Peninsula	2016	0.7303	0.8178	0.8258	0.9589

Table 7 illustrates that the generalized dimension in the second half period of the nationwide area and the urban agglomerations are greater than those in the first half, except for the urban agglomeration of Beijing-Tianjin-Hebei. Since the generalized dimension of the urban agglomerations of the Yangtze River Delta, Guangdong-Hong Kong-Macao Greater Bay Area, Chengdu-Chongqing, Middle Yangtze River, and Shandong Peninsula tends to be at or equal to 1 in the last half period, even if the time demarcations are different, it indicates that their high-speed railway network development tends to be in correspondence with the regional economic growth. It leads to a strengthening of adaptability between network development and economic growth. Interestingly, the generalized fractal dimension of the urban agglomeration of Beijing-Tianjin-Hebei before 2010 is 1.6872 > 1.0, indicating its economic growing rate in this period is distinctly higher than that of the high-speed railway network mileage argumentation. After 2010, however, the economic growth changed to significantly slower than the network development, with the generalized fractional dimension of 0.6180 < 1.0. It is contrary to the evolution trend of other regions and even is different from that of the whole country. It is worth noting that the network development, i.e., the increasing mileage in the early years of the urban agglomerations of Guangdong-Hong Kong-Macao Bay Area and Yangtze River Delta growing significantly faster than the increasing of GDP, i.e., the economic growth, as both generalized fractal dimensions were less than 1.0 (0.4764 and 0.3512). But after 2012 and 2013, the generalized fractal dimensions changed to 1.1871 and 1.0151 (larger than 1.0), respectively. Also, the network mileage growth rate in these two urban agglomerations is the most coordinated and mutually applicable to the increasing rate of GDP. Likewise, there is more coordinated development between the network development and economic growth in the urban agglomeration of Chengdu-Chongqing after 2013, with a generalized fractional dimension of 0.9234, nearly equal to 1.

## 4 Conclusion and future research

### 4.1 Conclusion

This study uses GIS and fractal theory and focuses on exploring and characterizing the spatial complexity, heterogeneity, and nonlinear structural characteristics of the high-speed railway networks in China, thus discussing the above aspects from the perspective of the nationwide area and urban agglomerations. Furthermore, the coordination between the high-speed railway network development and economic growth in China is distinguished distinctly based on the fractal models. The objective is to promote the optimization of the spatial structural organization pattern of the network and its coordinated development with the regional economy and thus to provide new points for the future planning and evolution of the high-speed railway networks in China. The results show that (1) the high-speed railway network in China has distinct fractal characteristics; (2) the network spatial structural organization shows inhomogeneity in the context of intensity, density, and complexity, especially with significant spatial heterogeneity; (3) in details, the length dimension of the network is generally higher than its branch dimension, and the network intensity is better than its complexity, thus it can be seen that the current structure of the network system is relatively simple, and its accessibility still has space to develop; (4) at the perspective of the nationwide area, the network development in the intersection area of core areas (IACA) is more relatively uniform, with the railway routes even in a small spatial range, nevertheless, from a regional perspective of urban agglomeration the uniformity of network coverage is still at a moderate level, particularly with the lowest coverage uniformity in the urban agglomeration of Guangdong-Hong Kong-Macao Bay Area; (5) both of the analysis of the coordinated relationships between the economic growth and high-speed railway network development in the nationwide area and urban agglomeration show with the network developing at a higher rate than the economic growing, but the increasing rate of both tending to be the same; (6) the urban agglomerations of Guangdong-Hong Kong-Macao Greater Bay Area and Yangtze River Delta are the regions where the growing rates of the network mileage are currently most coordinated and mutually applicable to the regional economic growth.

The finding of this study validates that spatial nonstationarity, heterogeneity, and complexity are pervasive in the evolution of high-speed railway networks in China. It expects that more pronounced spatial nonstationarity, heterogeneity, and complexity may emerge for high-speed railway networks in China in the process of rapid economic growth. Furthermore, this study gives the urban agglomerations in China a measure of comparability to high-speed railway networks, which different urban agglomerations can use to gauge the success of their urban development efforts. In summary, the results have shown that fractal dimensions, including length-radius dimension, branch-radius dimension, and Hausdorff dimension (similarity dimension), can offer better indications of the fractal property of high-speed railway networks in China. Compared to traditional Euclidean geometry modeling approaches, the fractal geometry model indicates better goodness of fit and effects by incorporating spatial nonstationarity and heterogeneity. It gives valuable insights into understanding the relationship between the structural characteristics of high-speed railway networks and the economic growth in China. It enables planners and decision-makers to make accurate predictions regarding the future coordinated development of high-speed railway networks with the rapid economic development and formulate more reasonable and scientific high-speed railway network planning strategies and policy measures.

### 4.2 Limitations and future research

Although the results derived from this paper have reached the research objectives, and have confirmed its hypotheses, there are still some limitations as follows.

1. It is worth noting that this study examined a positive correlation between high-speed railway network development and economic output. However, the relationships may vary with the function and development of the high-speed railway networks in different periods. For example, while new high-speed railways open in a region, the fractal characteristics of the region's high-speed railway network should change, thus reconstructing the relationship with regional economic growth. Therefore, future research should conduct a comparative analysis that indicates the different effects of the new high-speed railways opening and the original networks on economic growth.
2. This study reveals the impacts of economic growth on the fractal dimensions of the high-speed railway network in China. However, a region's human, land-use, and industrial activities are the main economic drivers, and relevant population, built-up density, and industrial indicators could play a vital role in influencing the fractal characteristics of high-speed railway networks. Therefore, future work should take the high-speed railway network to integrate the traditional railway networks as a whole research object and conduct more in-depth empirical analysis and comparison of different influencing factors affecting the fractal dimension to obtain more scientific and objective findings and gain a more comprehensive understanding and insight.
3. The length-radius dimension, branch-radius dimension, and Hausdorff dimension to characterize the structural characteristics of high-speed networks are widely used to provide a relatively easy and effective method for empirical estimation and exploration of non-self-similar objects. Nevertheless, the limitations include the variations in fractal dimensions for the same object when changing the measurement center, or the effect of grid resolution (Hausdorff dimension) and gyration radius difference (length-radius dimension and branch-radius dimension) on the value of fractal dimension. Therefore, future research should analyze the multifractal characteristics of the high-speed railway network in China to exhibit different fractal properties of the network at different scales and regions.

## Data availability statement

The data used to support the findings of this study are available from the corresponding author upon request. Furthermore, this study combines the high-speed railway routes and stations and other basic geographic data (including administrative divisions, POI (points of interest) and notes, etc.) from Basic Geographic Database of China with the proportion of 1:250,000 (China Geodetic Coordinate System 2000), which is provided by National Catalogue Service for Geographic Information authorized from National Geomatics Center of China (https://www.webmap.cn) and National Railway Construction and Planning Schematic of China (2021).

## Funding statement

This study was funded by Guangdong Basic and Applied Basic Research Foundation (Grant number 2021A1515011499), 10.13039/501100011788Philosophy and Social Science Planning Project of Guangdong Province (Grant number GD21CGL26), and Guangdong Basic and Applied Basic Research Foundation (Grant number 2023A1515010853).

## CRediT authorship contribution statement

**Xiaoshan Cai:** Formal analysis, Methodology, Validation, Writing – original draft, Writing – review & editing. **Shaopei Chen:** Conceptualization, Investigation, Methodology, Writing – original draft, Writing – review & editing, Supervision. **Xinying Lian:** Data curation, Validation.

## Declaration of competing interest

The authors declare that they have no known competing financial interests or personal relationships that could have appeared to influence the work reported in this paper.
